# Color-selective three-dimensional polarization structures

**DOI:** 10.1038/s41377-022-00961-y

**Published:** 2022-10-17

**Authors:** Yuttana Intaravanne, Ruoxing Wang, Hammad Ahmed, Yang Ming, Yaqin Zheng, Zhang-Kai Zhou, Zhancheng Li, Shuqi Chen, Shuang Zhang, Xianzhong Chen

**Affiliations:** 1 Institute of Photonics and Quantum Sciences, School of Engineering and Physical Sciences, Heriot-Watt University, Edinburgh, EH14 4AS UK; 2 Department of Mathematics and Physics, North China Electric Power University, Baoding, 071003 China; 3 School of Physics and Electronic Engineering, Changshu Institute of Technology, Suzhou, 215000 China; 4 State Key Laboratory of Optoelectronic Materials and Technologies, School of Physics, Sun Yat-sen University, Guangzhou, 510275 China; 5 School of Physics and TEDA Applied Physics Institute, Nankai University, 94 Weijin Road, Tianjin, 300071 China; 6 Department of Physics, University of Hong Kong, Hong Kong, China; 7 Department of Electronic & Electrical Engineering, University of Hong Kong, Hong Kong, China

**Keywords:** Metamaterials, Displays

## Abstract

Polarization as an important degree of freedom for light plays a key role in optics. Structured beams with controlled polarization profiles have diverse applications, such as information encoding, display, medical and biological imaging, and manipulation of microparticles. However, conventional polarization optics can only realize two-dimensional polarization structures in a transverse plane. The emergent ultrathin optical devices consisting of planar nanostructures, so-called metasurfaces, have shown much promise for polarization manipulation. Here we propose and experimentally demonstrate color-selective three-dimensional (3D) polarization structures with a single metasurface. The geometric metasurfaces are designed based on color and phase multiplexing and polarization rotation, creating various 3D polarization knots. Remarkably, different 3D polarization knots in the same observation region can be achieved by controlling the incident wavelengths, providing unprecedented polarization control with color information in 3D space. Our research findings may be of interest to many practical applications such as vector beam generation, virtual reality, volumetric displays, security, and anti-counterfeiting.

## Abstract (web summary)

A single metasurface is used to generate multiple 3D knots with predesigned polarization profiles. At a given region, only a single 3D polarization knot can be obtained for a single color. Different 3D polarization knots can be obtained by changing the incident colors (left). The generated polarization structures are unveiled after passing through a linear polarizer (right).

## Graphical abstract

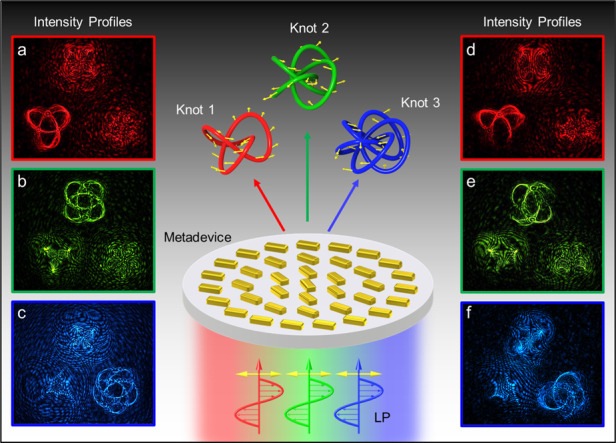

## Introduction

Polarization has been a central concept to our understanding of optics and has found many applications ranging from quantum science^1,2^ to our daily life (e.g., polarized sunglasses and 3D cinema). Polarization control has been used to record, process, and store information^3–6^. The ability to precisely control polarization distribution of light beam is critical to both fundamental science and practical applications. Conventional optical elements (e.g., polarizers, wave plates) for polarization manipulation typically treat polarization as a uniform characteristic of an optical beam that can be globally controlled. Light beams with spatially nonuniform polarization distributions have received great attention owing to their peculiar optical features including Möbius strips with non-orientable surfaces^7^, and their practical applications such as higher-resolution lithography^8^ and patterning of lyotropic chromonic liquid crystals by photoalignment^9^. There has been much advancement in the theoretical understanding of 3D polarization structures to further our knowledge^10,11^, but the experimental research has not advanced at the same rate. This is essentially due to the technical and fundamental challenges in creating 3D polarization profiles with conventional methods. So far, only a few types of these structures were generated at the expense of complex system, large volume, and high cost^7,12,13^, limiting their practical applications.

Optical metasurfaces, planar nanostructured interfaces, have attracted increasing interests due to their unprecedented capability in light manipulation at subwavelength scale^14–24^. The optical metasurface-based flat optics has revolutionized design concepts in photonics, providing a compact platform to develop ultrathin (light wavelength scale) and lightweight planar optical devices with novel functionalities that cannot be obtained with conventional optical elements, with examples including dual-polarity lenses^15^, multi-foci lenses^20^, light sward lenses^21^, and polarization sensitive holograms^17–19,22–25^. Recently, 3D polarization knots^26^ and longitudinally variable polarization^27^ were experimentally demonstrated. Multispectral polarization manipulation will add more degrees of freedom. Furthermore, multifunctional optical devices have profound implications for the research fields where lightweight and integrated optical systems are highly desirable. There is an urgent need to develop multifunctional ultrathin devices that can simultaneously encode color and intensity information into 3D polarization profiles. However, there has been no report on engineering 3D polarization structures with predesigned intensity and color information.

To tackle the above-mentioned challenges, we aim to create multiple 3D polarization structures, together with engineered intensity and color information, by developing novel ultrathin optical devices using optical metasurfaces. Here we experimentally demonstrate a single metasurface device that can change a linearly polarized (LP) incident light beam into multiple 3D knots with different predesigned polarization states along the light propagation direction. In the same observation region, different polarization structures are realized by controlling the incident wavelengths. The flexible and controllable generation of 3D polarization distributions with customized intensity profiles of different colors (wavelengths) may open a door to many practical applications such as vector beam generation, virtual reality, color displays, information security, anti-counterfeiting, and high-density information storage.

## Results

**Fig. 1:**
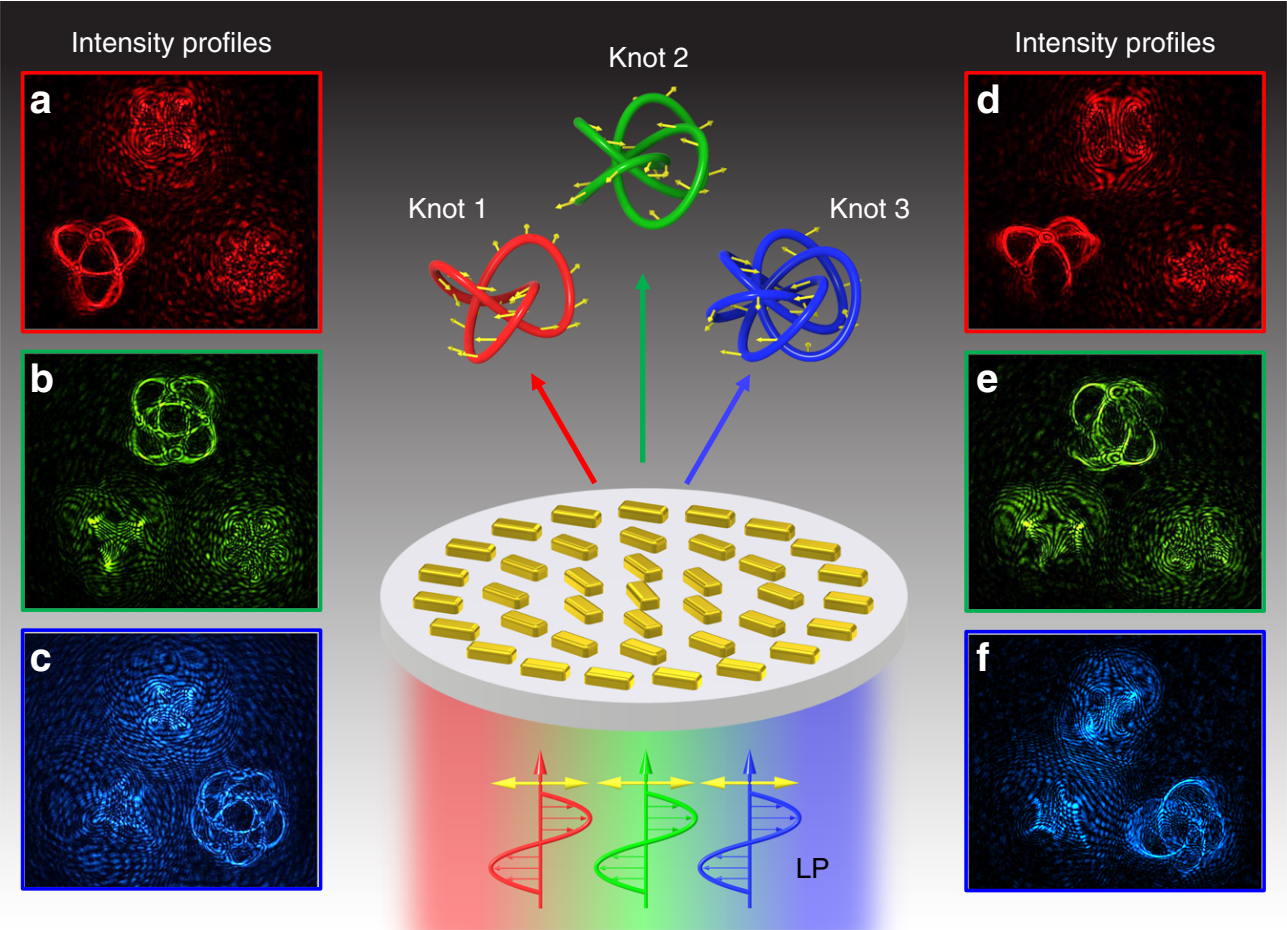
Schematic of the metasurface device for generating color-selective 3D polarization structures. Here, a 3-foil knot (knot 1), a 4-foil knot (knot 2), and a 5-foil knot (knot 3) are selected as complex polarization structures. Knots 1, 2, and 3 are predesigned polarization structures in the same observation region for the wavelengths of 650, 575, and 500 nm, respectively. The polarization directions of points on the 3D knots are denoted with yellow arrows. Both polarization and color information (wavelength) of these three knots are encoded into a single metasurface consisting of gold nanorods. **a**–**c** The measured intensity profiles upon the illumination of a horizontal LP light beam before and **d**–**f** after passing through a polarizer (analyzer). **a** and **d** are generated at *λ* = 650 nm, **b** and **e** at *λ* = 575 nm, and **c** and **f** at *λ* = 500 nm in the same observation plane of *z* = 500 μm. The intensity profiles **d**–**f** correspond to the analyzer with a transmission axis along the vertical direction

Figure 1 shows the schematic of our proposed metasurface device (metadevice) for the generation of multiple 3D polarization structures. The metadevice consists of gold nanorods with spatially variant orientations sitting on a glass substrate. Upon the illumination of a LP beam, multiple 3D knots with predesigned polarization profiles are created. At a given observation region of interest, only a single 3D knot can be obtained for a single color (wavelength). Different 3D polarization knots can be obtained by changing the incident wavelengths (Fig. 1a–f). The generated polarization structures are unveiled after passing through a linear polarizer, leading to various modulated intensity patterns captured by a charge-coupled device (CCD) as depicted in Fig. 1d–f.

To create multiple 3D polarization knots with the engineered polarization profiles and color information, the design of such a metadevice involves multi-foci design, polarization rotation, and color multiplexing in 3D space. We start from a metalens model with multiple focal points. A continuous 3D focal curve is obtained when the density of focal points is dramatically increased. Then, we add the polarization information for each focal point, which is realized based on the polarization rotation functionality of the metalens upon the illumination of incident LP light. The metalens can simultaneously focus LP light and rotate its polarization direction at each focal point with a predesigned rotation angle^20^. The polarization rotation at each focal point is realized based on the superposition of two circular polarization states, which are controlled by the same geometric metasurface. Finally, multiple polarization structures with different wavelengths are added in the design. Each wavelength corresponds to a specific polarization structure. The color-selective functionality is based on the dispersion effect of the metalens, whose focal length varies with the incident wavelength. Although the multiple polarization structures simultaneously exist for any incident wavelength, they can be designed to be located at different positions. In the same observation region, only a single polarization structure is obtained upon the illumination of a light beam with a single wavelength.

### Design of multi-foci metasurface

To design an arbitrary multi-foci metalens in 3D space, we first formulate the phase distribution of the lens for the *n*th focal point, which is given by

1 $$\varphi \left( {x,y} \right)_n = - \frac{{2\pi }}{\lambda }\left( {\sqrt {f_n^2 + \left( {x - u_n} \right)^2 + \left( {y - v_n} \right)^2} - \sqrt {f_n^2 + u_n^2 + v_n^2} } \right)$$

where *λ* is the operating wavelength, *f* is the focal length, *u* and *v* are the locations of a focal point along the *x* and *y* directions, respectively. (*u*, *v*, *f*) are the 3D coordinates of the focal point. The position of a focal point (on the optical axis or off-axis) depends on the values of *u* and *v*. The desired field profile of the multi-foci lens is the sum of the fields for generating each focal point, and the corresponding phase distribution Φ(*x*, *y*) is given by

2 $${{\Phi }}\left( {x,y} \right) = \arg \left\{ {\mathop {\sum}\limits_{n = 1}^N {e^{i\varphi (x,y)_n}} } \right\}$$

where *N* is the total number of the desired focal points and *n* ranges from 1 to *N* (integer numbers). A geometric metasurface is used to realize the designed metalens, which can produce Pancharatnam–Berry phase profiles associated with the handedness of the circularly polarized (CP) light, i.e., left circularly polarized (LCP) and right circularly polarized (RCP) light^28–30^. By controlling the orientation angles *θ* of the individual nanorods, one can obtain the desired phase profile. The local abrupt phase change is Φ = ±2*θ*, where “+” and “−” represent the sign of the phase change for the incident LCP and RCP light beams, respectively.

### Adding polarization rotation functionality

The multiple focal points in Eq. (2) can only be generated for one CP light beam, while the opposite circular polarization would generate a divergent lensing effect due to the flipped sign of the phase profile^15^. To simultaneously focus an LP light beam and control its polarization direction at each focal point, a metadevice is required to focus both LCP and RCP light beams, but with different phases. The phase distribution is governed by

3 $${{\Phi }}\left( {x,y} \right) = \arg \left\{ {\mathop {\sum}\limits_{n = 1}^N {\left( {e^{i\varphi (x,y)_n} + e^{ - i\varphi (x,y)_n}} \right)} } \right\}$$

Where the two terms $e^{i\varphi (x,y)_n}$ and $e^{ - i\varphi (x,y)_n}$ are respectively responsible for the focusing an LCP light beam and an RCP one. With an LP incident beam, the superposition of the LCP and RCP light beams with equal intensity at the same focal point can generate polarization rotation. Thus, the polarization direction (*ϕ*_*n*_) for each focal point in Eq. (3) can be precisely controlled with the following phase distribution of the metadevice

4 $${{\Phi }}\left( {x,y} \right) = \arg \left\{ {\mathop {\sum}\limits_{n = 1}^N {\left( {e^{i\left[ {\varphi (x,y)_n + \phi _n} \right]} + e^{ - i\left[ {\varphi (x,y)_n - \phi _n} \right]}} \right)} } \right\}$$

For an LP incident beam, the overall generated output beam consists of four CP components with different phases: $A_{RCP} \cdot e^{i\left[ {\varphi (x,y)_n + \phi _n} \right]}$, $A_{RCP} \cdot e^{ - i\left[ {\varphi (x,y)_n - \phi _n} \right]}$, $A_{LCP} \cdot e^{ - i\left[ {\varphi (x,y)_n + \phi _n} \right]}$, and $A_{LCP} \cdot e^{i\left[ {\varphi (x,y)_n - \phi _n} \right]}$, where *A*_*RCP*_ and *A*_*LCP*_ correspond to the amplitudes of RCP and LCP light. Among these four components, $A_{RCP} \cdot e^{i\left[ {\varphi (x,y)_n + \phi _n} \right]}$ and $A_{LCP} \cdot e^{i\left[ {\varphi (x,y)_n - \phi _n} \right]}$ contribute to the construction of each focal point with predesigned polarization rotation.

### Color multiplexing

To create wavelength-multiplexed polarization profiles, the phase profile of the metadevice is given by

5 $${{\Phi }}\left( {x,y} \right) = \arg \left\{ {\mathop {\sum}\limits_{m = 1}^M {\mathop {\sum}\limits_{n = 1}^N {\left( {e^{i\left[ {\varphi \left( {x,y} \right)_{m,n} + \phi _{m,n}} \right]} + e^{ - i\left[ {\varphi \left( {x,y} \right)_{m,n} - \phi _{m,n}} \right]}} \right)} } } \right\}$$

where $\varphi (x,y)_{m,n} = - \frac{{2\pi }}{{\lambda _m}}\left( {\sqrt {f_{m,n}^2 + \left( {x - u_{m,n}} \right)^2 + \left( {y - v_{m,n}} \right)^2} - \sqrt {f_{m,n}^2 + u_{m,n}^2 + v_{m,n}^2} } \right)$, *n* and *m* represent the *n*th focal point on the *m*th polarization structure, *λ*_*m*_ is the wavelength for the *m*th structure. *N* and *M* are the total numbers of the focal points and 3D polarization structures, respectively. (*u*_*m*,*n*_, *v*_*m,n*_, *f*_*m,n*_) are the coordinates of a given point with a polarization rotation angle of *ϕ*_*m,n*_ on the created 3D polarization structures. Going beyond previous works^26,27^, this design features color-selective functionality and multiple 3D polarization structures. In a given observation region, different 3D polarization structures can be generated by controlling the operating wavelengths.

As a proof of concept, a 3-foil knot (knot 1, *m* = 1), a 4-foil knot (knot 2, *m* = 2), and a 5-foil knot (knot 3, *m* = 3) are selected as the polarization knots. The coordinates of each point on the 3D knots are given by the following parametric equations:

6 $$\left\{ {\begin{array}{*{20}{l}} {u_{m,n} = a\left( {\sin \phi _{m,n} + 2\sin \left[ {(1 + m)\phi _{m,n}} \right]} \right) + r\cos {\it{\Lambda }}_m} \\ {v_{m,n} = a\left( {\cos \phi _{m,n} - 2\cos \left[ {\left( {1 + m} \right)\phi _{m,n}} \right]} \right) + r\sin {\it{\Lambda }}_m} \\ {f_{m,n} = - 1.5a\sin \left[ {\left( {2 + m} \right)\phi _{m,n}} \right] + f_0} \end{array}} \right.$$

**Fig. 2:**
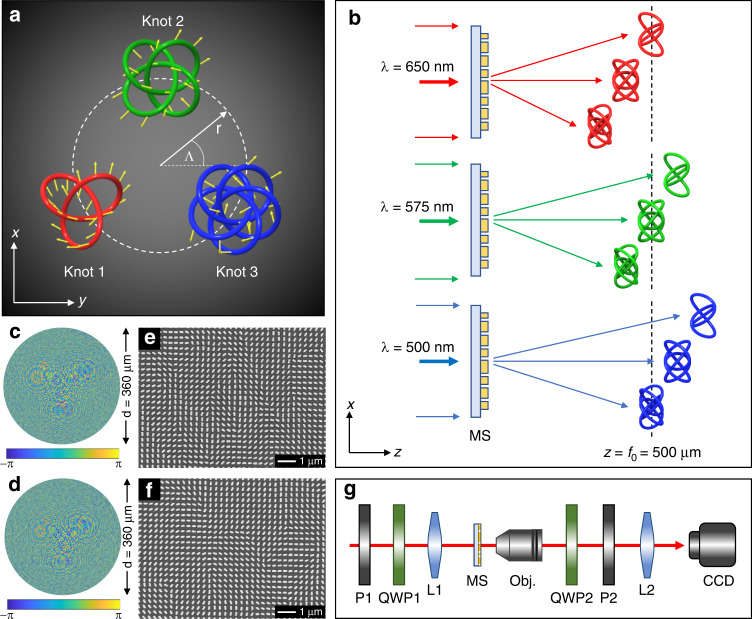
Polarization knots arrangement, color-selective mechanism, SEM images of fabricated samples, and schematic of the experimental setup. **a** The arrangement of the 3D polarization knots. The polarization direction of any given point is denoted with yellow arrows. **b** Color-selective mechanism. The locations of three different 3D knots are given upon the illumination of the incident light at *λ* = 650 nm (top), *λ* = 575 nm (middle), and *λ* = 500 nm (bottom), respectively. The observation region is defined by the two planes: *z* = 485 μm and *z* = 515 μm. The plane *z* = 500 μm is the middle plane of the observation region. Only one 3D polarization knot is obtained for a single incident wavelength; thus, the color-selective functionality is realized. **c** and **d** are the desired phase profiles for the two metadevices that can respectively generate multiple polarization knots and color-selective polarization knots in the same observation region. The corresponding scanning electron microscope (SEM) images of the fabricated metasurface devices are shown in (**e**) and (**f**), respectively. **g** Schematic of the experimental setup for characterizing the developed metadevices. P1 and P2: polarizers, QWP1 and QWP2: quarter wave plates, L1: convex lens (*f* = 15 cm), MS: metasurface device, Obj.: ×20 objective lens, L2: convex lens (*f* = 10 cm), CCD: charge-coupled device

Where *r* cos *Λ*_*m*_ and *r* sin *Λ*_*m*_ are the locations of the knot *m* in the *xy* plane with a radius of *r* from the center of a focal plane and an angle of *Λ*_*m*_ with respect to (w.r.t.) the *x* axis (Fig. 2a). *a* is a constant number used to define the knot dimensions. The polarization direction *ϕ*_*m,n*_ of any point *n* on the knot *m* is denoted with a yellow arrow. The 3D knots extend into the *z* direction and the plane *z* = *f*_0_ is the middle observation plane of the knots.

In the design, *a* is defined as 10 μm, *f*_0_ is 500 μm, and *r* is 50 μm. The orientation angles *Λ*_*m*_ of the knots are 210°, 90°, and −30° for *m* = 1, 2, and 3, respectively. The polarization rotation angles of the points on each knot vary from 0 to 2π. In order to maintain the same distance between two adjacent points for different knots, the knots are designed to have different numbers of the total points *N*, which are 2000, 2860, and 3728 points for knots 1, 2, and 3, respectively.

The color-selective functionality is inspired by the dispersion effect of a metalens, whose focal length varies with the wavelengths. First, we design a metadevice that can simultaneously generate three polarization structures in the same observation region described by Eq. (5) at a single operation wavelength (*λ*_*m*_) of 650 nm. Then the wavelength information (*λ*_1_ = 650 nm, *λ*_2_ = 575 nm, and *λ*_3_ = 500 nm) is added to the polarization profiles to provide the color-selective functionality for the second metadevice design. The observation region is defined as the 3D space between two planes *z* = 485 μm and *z* = 515 μm, and the middle observation plane of the 3D knot is located at *z* = 500 μm. When the metadevice is illuminated by LP light at *λ* = 650 nm (red), three polarization knots are generated, but only knot 1 is located in the observation region and the other two knots are outside this region (Fig. 2b, top). When the incident wavelength is 575 nm (green), knot 2 is located in the observation region while knot 1 and knot 3 are outside this region (Fig. 2b, middle). Similarly, only knot 3 is in the observation region at *λ* = 500 nm (blue) (Fig. 2b, bottom). Therefore, different polarization structures can be obtained by controlling the wavelengths of the incident light. The calculated phase profiles of the first and second metadevices are shown in Fig. 2c, d, respectively.

The designed metadevices consist of gold nanorods with spatially variant orientation sitting on a glass substrate. The orientation angles *θ*(*x*, *y*) of the nanorods are defined by Φ(*x*, *y*)/2. Each nanorod is 220 nm long, 130 nm wide, and 40 nm thick. The lattice constant is 300 nm along both *x* and *y* directions. The designed metasurfaces can operate in the whole visible region^26,31^ and their response spectrum is provided in Supplementary Section 1. The designed devices are fabricated based on the standard electron-beam lithography, followed by the electron-beam deposition and the lift-off process. The diameter of the fabricated circular sample is 360 μm. Figure 2e, f shows the scanning electron microscope images of the fabricated metadevices for creating multiple polarization knots and color-selective polarization knots in the same observation region, respectively. The diagram of an experimental setup to characterize the fabricated metadevices is shown in Fig. 2g and the details are in the Materials and methods section. Here, all the simulated 3D structures are obtained based on the Fresnel–Kirchhoff diffraction integral^32^.

**Fig. 3:**
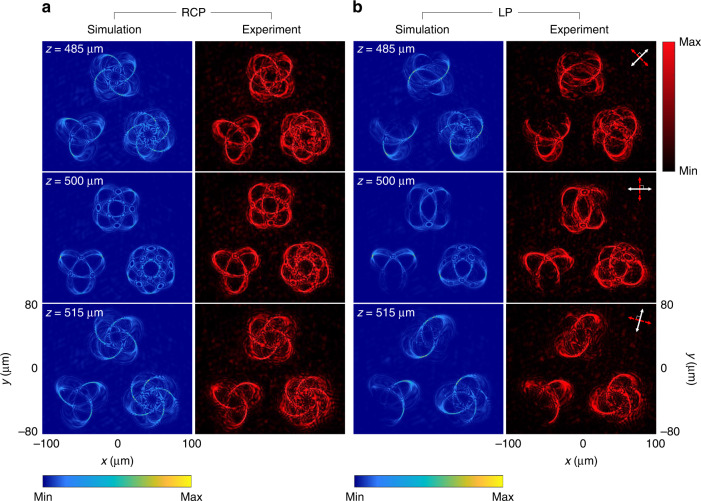
Simulation and experimental results of the generated multiple 3D polarization knots at *λ* = 650 nm. **a** The intensity distributions at different observation planes under the illumination of RCP light beam. **b** The intensity distributions under the illumination of an LP light beam for polarization distribution detection with different transmission axes of the linear polarizer and the analyzer at different observation planes. The simulated (left) and measured intensity profiles (right) at three observation planes after passing through the analyzer by considering Malus’ law with different combinations of the transmission axes of the polarizer and the analyzer. The white and red double arrows indicate the transmission axes of the first polarizer (P1) and the analyzer (P2), respectively. P1⊥P2. The direction of the white arrows: 45° (*z* = 485 μm), 0° (*z* = 500 μm), and 75° (*z* = 515 μm) w.r.t. the *x* axis

Figure 3 shows the simulation and experimental results of the generated multiple 3D polarization knots at *λ* = 650 nm in the three observation planes (*z* = 485 μm, 500 μm, and 515 μm). In this design, all 3D knots are simultaneously created at the same observation region under the illumination of the RCP light beam (Fig. 3a). The intensity patterns of 3D knots are clearly observed. To generate 3D polarization profiles, the incident LP light beam is used. In the observation planes, the intensity distribution is modulated based on Malus’ law where the minimum intensity corresponds to the positions where the predesigned polarization rotation is equal to 2*α* and 2*α*+π (*α* is the polarization direction of the incident LP light beam w.r.t. the *x* axis). For instance, for the LP light beam with a polarization direction of 45°, intensity gaps can be seen at the predesigned positions with polarization rotation angles of 90° and 270° (see Fig. 3b at *z* = 485 μm). For *z* = 500 and 515 μm, they are found at the angles α of 0° and 75°, respectively (Fig. 3b). The projection of the 3D polarization knots on the given observation planes are in good agreement with the simulated results. To show the robustness of our design, we also develop a metadevice that can simultaneously create five 3D polarization knots at a single operating wavelength (see Supplementary Section 3).

**Fig. 4:**
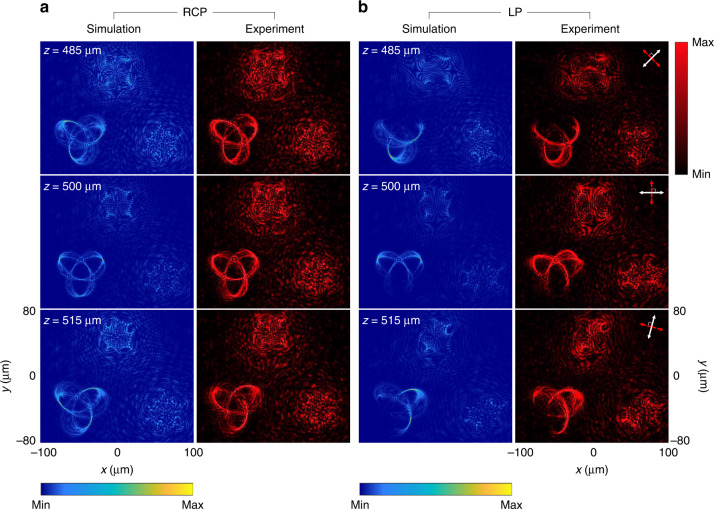
Simulation and experimental results of the selected 3D polarization knots at *λ* = 650 nm. **a** The intensity distributions at different observation planes upon the illumination of RCP light. **b** The intensity distributions at different observation planes upon the illumination of an LP light beam for polarization distribution detection with different combinations of transmission axes of the linear polarizer and the analyzer. The direction of the white arrows: 45° (*z* = 485 μm), 0° (*z* = 500 μm), and 75° (*z* = 515 μm) w.r.t. the *x* axis

**Fig. 5:**
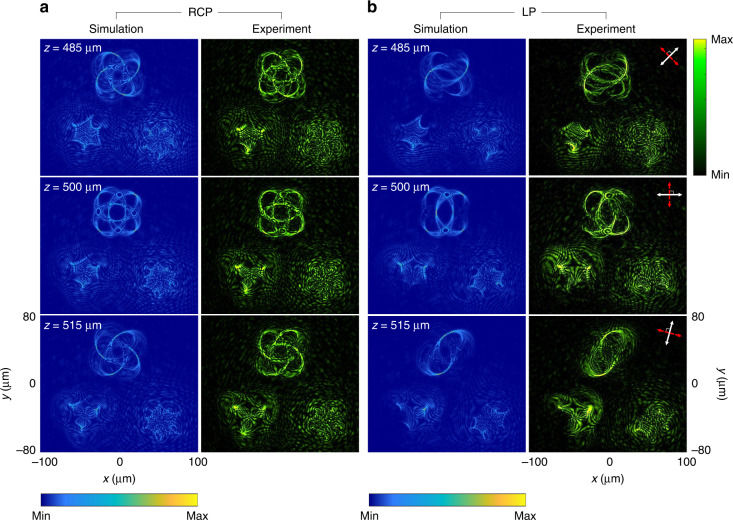
Simulation and experimental results of the selected 3D polarization knots at *λ* = 575 nm. **a** The intensity distributions at different observation planes upon the illumination of RCP light. **b** Polarization structure detection with the same detection method as that in Fig. 4b

**Fig. 6:**
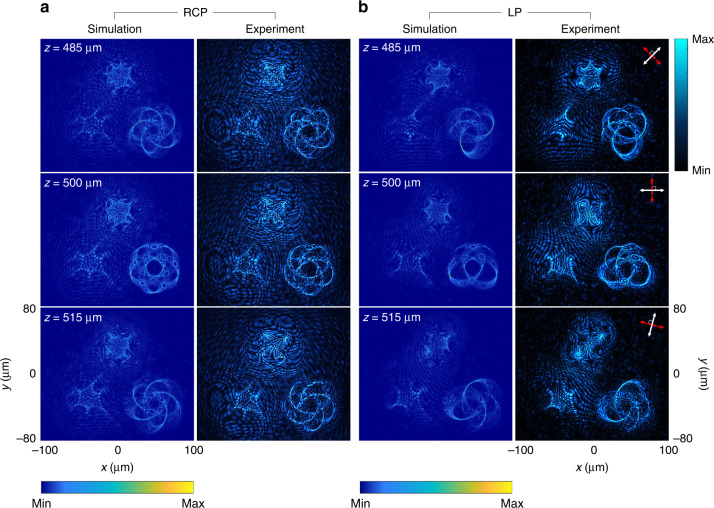
Simulation and experimental results of the selected 3D polarization knots at *λ* = 500 nm. **a** The intensity distribution at different observation planes upon the illumination of RCP light. **b** Polarization structure detection with different combinations of transmission axes of the first polarizer and the second polarizer

We then characterize the metasurface for generating color-selective 3D polarization knots by changing the incident wavelengths. Figure 4 shows the created 3D polarization knots when the incident wavelength is 650 nm. When the polarization state of the incident beam is RCP, knot 1 can be clearly seen from the three observation planes (i.e., *z* = 485, 500, and 515 μm) while the other two knots are blurred and unrecognizable (Fig. 4a). With an incident LP light beam, the intensity gaps are found on those three observation planes when the combinations of transmission axes of the P1 and P2 are (45°, 135°), (0°, 90°), and (75°, 165°), respectively (Fig. 4b). In Fig. 5a, b, knot 2 and knot 3 on the observation plane are obtained by tuning the operating wavelength to 575 and 500 nm, respectively (Fig. 6a, b). The locations of all generated 3D knots for each operation wavelength are provided in Supplementary Section 4. The evolution process of the generated 3D polarization structures along the light propagation direction with the incident RCP light beam can be seen clearly by gradually changing the observation plane (Supplementary Movie 1). The experimental results unambiguously show that the metadevice can generate the 3D polarization knots as predicted by the theoretical analysis. Simulation results of the design with other different types of knots are provided in Supplementary Section 5.

## Discussion and conclusion

We have experimentally demonstrated a multifunctional metasurface device that can enable the construction of color-selective 3D polarization structures. Simulated and experimental results of the conversion efficiency are provided in Supplementary Section 1. To verify the design, we use a metasurface consisting of gold nanorods with a low conversion efficiency, which can be dramatically increased by using dielectric metasurfaces^33,34^. Unlike the previous work^26^, where the metasurface design was based on a single wavelength and a single 3D polarization structure, multiple wavelengths, and multiple 3D polarization structures are included in this design. The color-selective functionality is realized based on the combination of the unique design and the dispersion effect of the metasurface. Benefiting from the new design, different 3D polarization structures are realized in the same observation region by controlling the wavelengths of the incident light. In contrast, the polarization structure cannot be changed and the developed metadevice has no color-selective functionality in our previous work. The maximum number of wavelengths in the color-selective functionality in this work is three, which can be increased by decreasing the sizes of the polarization structures along the light propagation direction or by using metasurfaces consisting of nanostructures with different feature sizes (e.g., length, width, and height). Our work may find more potential applications in optics and fundamental science. The color information in the polarization structure can dramatically increase the information capacity, which can perform extremely challenging tasks that are not possible with conventional optics. The proposed method can be used not only to realize the color-selective 3D polarization structure generation, but also to realize a 3D polarization structure with multiple colors. For example, multiple colors can be included in the same 3D polarization knot. Simulation results for the same polarization knot with different color and polarization distributions are provided in Supplementary Section 6. Color mixing functionalities for the same polarization structures can also be realized by using a superpixel consisting of multiple dielectric nanopillars with different feature sizes^35^. The capability of simultaneously encoding color and intensity information into 3D polarization profiles can realize “3D color image hidden in 3D color image”. The color image formed by the 3D polarization structure with the predesigned color and intensity distributions can be changed to another color image (hidden image) based on Malus’ law with the aid of a polarizer.

Our design method combines color information and 3D polarization manipulation, offering more degree of freedom for polarization engineering and enabling the realization of wavelength selective functionality. The unique properties of the developed metasurface devices may promote both fundamental research (e.g., complex 3D polarization structures) and practical applications (e.g., virtual reality and anti-counterfeiting). Furthermore, the developed metasurface devices can be vertically integrated to build a complex system composed of various planar components (e.g., gratings, splitters) to perform sophisticated tasks. We expect that this capability will fuel the continuous progress of wearable and portable consumer electronics and optics where low-cost and miniaturized systems are in high demand.

## Materials and methods

### Device fabrication

The plasmonic metasurfaces consist of gold nanorods with spatially variant orientations sitting on an ITO-coated glass substrate. First, the ITO-coated substrate is cleaned with acetone for 10 min and isopropyl alcohol (IPA) for 10 min in an ultrasonic bath. Then, the substrate is rinsed in deionized water and dried with a nitrogen gun. The positive poly methyl methacrylate (PMMA) 950 A2 resist is spin coated on the SiO_2_ layer at 1000 rpm for 60 s followed by 1500 rpm for 15 s, producing a 100-nm-thick PMMA. After that, the sample is baked on a hotplate at 180 °C for 5 min. The electron-beam lithography (Raith PIONEER, 30 KV) is used to define nanopatterns in the PMMA film. The sample is developed in MIBK:IPA (1:3) for 45 s followed by the stopper (IPA) for 45 s. A gold film (40 nm) is deposited on the sample using an electron-beam evaporator. Finally, the metasurfaces (see Fig. 2e, f and Supplementary Fig. S2c) are fabricated after the lift-off process in acetone.

### Experimental setup

The diagram of an experimental setup to characterize the fabricated metadevices is shown in Fig. 2g. A light beam with tunable wavelengths is generated by a supercontinuum laser source (NKT Photonics SuperK EXTREME). Polarization states of the generated beam can be controlled by using a linear polarizer (P1) and a quarter wave plate (QWP1) in front of the metadevice. A convex lens (L1) is used to weakly focus the light beam onto the metadevice. An objective lens with a magnification of ×20, a convex lens (L2), and a CCD camera are used to collect the output light and image the 3D knots for visualization. The objective lens is mounted on a motorized translation stage, which allows us to obtain the intensity distributions of the created 3D knots along the *z* direction. Another pair of a quarter wave plate (QWP2) and a linear polarizer (P2) behind the objective lens is used to filter out the unconverted part of the transmitted light. To characterize the metadevice with an incident LP light beam, both QWP1 and QWP2 are removed, and the transmission axes of the P1 and P2 are kept perpendicular to each other. The details of the experimental setup are explained in Supplementary Section 2.

### Supplementary information

Conversion efficiency of the plasmonic metasurfaces, details of the experimental setup, metadevice to create five 3D polarization knots at a single operation wavelength, locations of created 3D knots at different wavelengths, a movie clip to show the evolution process of 3D polarization knots along the light propagation direction.

## Supplementary information

Supplementary Information

Evolution of generated 3D polarization structures along the light propagation direction
