# Supplementary material for: Color-selective three-dimensional polarization structures
Source: Light Sci Appl. 2022 Oct 17;11:302. doi: 10.1038/s41377-022-00961-y (PMC9576785; doi:10.1038/s41377-022-00961-y)
Supplement: Supplementary file 1 — Supplementary Information [file 41377_2022_961_MOESM1_ESM.docx]

Supplementary Information for Color Selective Three-Dimensional Polarization Structures

**Yuttana Intaravanne^1^, Ruoxing Wang^2^, Hammad Ahmed^1^,** **Yang Ming^3^, Yaqin Zheng^4^,** **Zhang-Kai Zhou^4^,** **Zhancheng Li^5^, Shuqi Chen^5^, Shuang Zhang^6,7,†^, and Xianzhong Chen^1,*^**

*1. Institute of Photonics and Quantum Sciences, School of Engineering and Physical Sciences, Heriot-Watt University, Edinburgh, EH14 4AS, UK*

*2. Key Laboratory of In-Fiber Integrated Optics of Ministry of Education, College of Physics and Optoelectronic Engineering, Harbin Engineering University, Harbin, 150001, China*

*3. School of Physics and Electronic Engineering, Changshu Institute of Technology*

*Suzhou, 215000, China*

*4. State Key Laboratory of Optoelectronic Materials and Technologies, School of Physics, Sun Yat-sen University, Guangzhou, 510275, China*

*5. School of Physics and TEDA Applied Physics Institute, Nankai University, 94 Weijin Road, Tianjin, 300071, China*

*6. Department of Physics, University of Hong Kong, Hong Kong, China*

*7. Department of Electronic & Electrical Engineering, University of Hong Kong, Hong Kong, China*

**†** Email: [shuzhang@hku.hk](mailto:shuzhang@hku.hk)

* Email: [x.chen@hw.ac.uk](mailto:x.chen@hw.ac.uk)

**Supplementary Section 1.** **Conversion efficiency of transmissive metasurfaces**

The plasmonic metasurfaces consist of gold nanorods with spatially variant orientations sitting on a glass substrate. The fabricated nanorods are about 220 nm long, 130 nm wide, and 40 nm high. The simulation results are obtained by using the frequency domain solver of the Computer Simulation Technology (CST) Microwave Studio software. The refraction index of the glass substrate is 1.46. The unit cell boundary is used along the *x* and *y* directions, and the open boundary is used along the *z* direction. The simulated and experimental results are provided in Figure S1. The difference between simulation and experiment is mainly due to the imperfection of the fabricated samples.


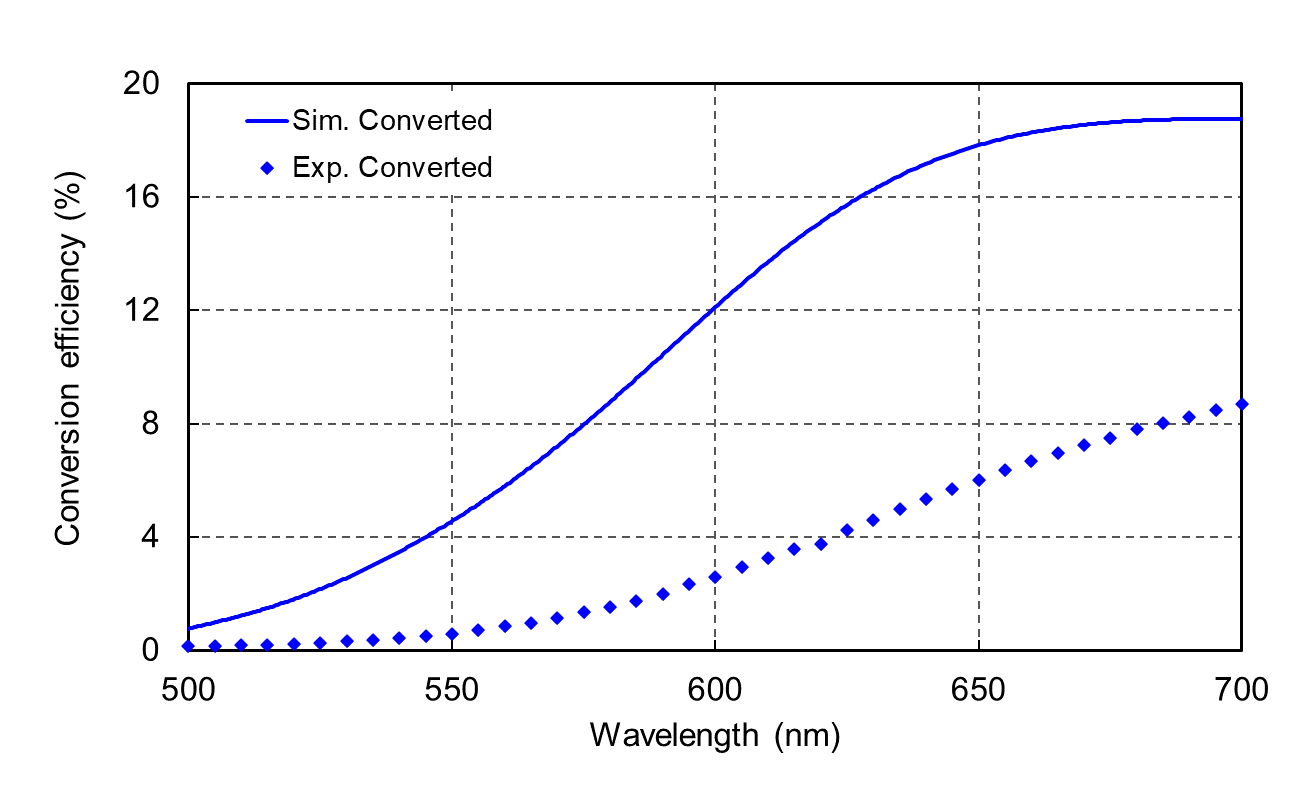


**Figure S1** Simulated and measured conversion efficiencies.

Although the conversion efficiency at the resonant wavelength of plasmonic metasurface is high, we have to consider the conversion efficiencies at three different wavelengths. To ensure there are no big changes in the conversion efficiencies at different wavelengths, it would be better to avoid the resonant wavelength.

**Supplementary Section 2. Working principle of the experimental setup**

The Jones vectors and Jones matrices are used to represent the polarization state of the light beams and the functionality of the optical elements. Each nanorod in the plasmonic metasurface can be considered as the combination of a perfect polarizer (low percentage) and a piece of normal flat glass slab, $\left[ \begin{matrix} 1 & 0 \\ 0 & 1 \end{matrix} \right]$. Thus, the Jones matrix of each nanorod ($J_{nanorod})$ can be written as

$J_{nanorod}=A\left[ \begin{matrix} 1 & 0 \\ 0 & 1 \end{matrix} \right]+B\left[ \begin{matrix} \cos^{2} \theta& \sin\theta\cos\theta\\ \sin\theta\cos\theta& \sin^{2} \theta\end{matrix} \right]$ (S1)

where *θ* is an orientation angle of each nanorod with respect to (w.r.t.) the *x* axis. *A* and *B* are the coefficients of the conversion efficiency of that glass slab and nanorods, respectively. In Figure 2g in the main text, the incident light beam ($E_{in})$ is generated by the polarizer P1 with the transmission axis along the *x* direction. Thus, its Jones vector is given by $E_{in}=\left[ \begin{matrix} 1 \\ 0 \end{matrix} \right]$. The polarization state of the $E_{in}$ can be changed by using the quarter waveplate QWP1, whose Jones matrix is given by

$J_{QWP1}=e^{-\frac{i\pi}{4}}\left[ \begin{matrix} \cos^{2} \beta_{1}+i\sin^{2} \beta_{1} & \left( 1-i \right)\sin\beta_{1}\cos\beta_{1} \\ \left( 1-i \right)\sin\beta_{1}\cos\beta_{1} & \sin^{2} \beta_{1}+i\cos^{2} \beta_{1} \end{matrix} \right]$ (S2)

where $\beta_{1}$ is the orientation angle of the fast axis w.r.t. the *x* direction. When the $E_{in}$ passes through the QWP1 with the angle $\beta_{1}$ of $\frac{\pi}{4}$, the output vector can be calculated by

$J_{QWP1}E_{in}=e^{-\frac{i\pi}{4}}\left[ \begin{matrix} \cos^{2} \frac{\pi}{4}+i\sin^{2} \frac{\pi}{4} & \left( 1-i \right)\sin\frac{\pi}{4}\cos\frac{\pi}{4} \\ \left( 1-i \right)\sin\frac{\pi}{4}\cos\frac{\pi}{4} & \sin^{2} \frac{\pi}{4}+i\cos^{2} \frac{\pi}{4} \end{matrix} \right]\left[ \begin{matrix} 1 \\ 0 \end{matrix} \right]=\frac{1}{\sqrt{2}}\left[ \begin{matrix} 1 \\ -i \end{matrix} \right]$ (S3)

Here, a right circularly polarized (RCP) light beam is generated. When the RCP beam passes through a nanorod, the Jones vector of the transmitted beam can be written as

$J_{nanorod}RCP=\frac{2A+B}{2\sqrt{2}}\left[ \begin{matrix} 1 \\ -i \end{matrix} \right]+\frac{B}{2\sqrt{2}}e^{-i2\theta}\left[ \begin{matrix} 1 \\ i \end{matrix} \right]$ (S4)

The transmitted light includes two main parts, non-converted part: $E_{nc}=\frac{2A+B}{2\sqrt{2}}\left[ \begin{matrix} 1 \\ -i \end{matrix} \right]$, and converted parts: $\frac{B}{2\sqrt{2}}e^{-i2\theta}\left[ \begin{matrix} 1 \\ i \end{matrix} \right]$. The non-converted part has the same polarization state as that of the incident beam, while the converted part has an opposite helicity and an additional phase shift $-2\theta$ generated by the geometric metasurface. By controlling the orientation angle $\theta$ of the nanorod in each unit cell, the phase shift can be tuned, ranging from $0$ to $2\pi$.

**2.1 Filtering the non-converted part from the incident RCP light beam**

To filter out the non-converted part, a pair of a quarter waveplate (QWP2) and a linear polarizer (P2) is used. The Jones matrix of the QWP2 is the same as whose QWP1, but the fast axis is defined as $\beta_{2}$. The Jones matrix of the P2 ($J_{P2})$ is given as

$J_{P2}=\left[ \begin{matrix} \cos^{2} \delta& \sin\delta\cos\delta\\ \sin\delta\cos\delta& \sin^{2} \delta\end{matrix} \right]$ (S5)

where $\delta$ is an angle of the transmission axis of the P2 w.r.t. the *x* axis. In this case, the angle $\beta_{2}$ is set parallelly to $\beta_{1}$, which is $\frac{\pi}{4}$. The angle $\delta$ is set to 0 which is parallel to whose P1. After the non-converted part ($E_{nc}$) passing through the P2, it can be filtered out as follows

$J_{P2}J_{QWP2}\frac{2A+B}{2\sqrt{2}}\left[ \begin{matrix} 1 \\ -i \end{matrix} \right]=\left[ \begin{matrix} 0 \\ 0 \end{matrix} \right]$ (S6)

After the converted part ($E_{c}$) passing through the P2, the output vector can be written as

$J_{P2}J_{QWP2}\frac{B}{2\sqrt{2}}e^{-i2\theta}\left[ \begin{matrix} 1 \\ i \end{matrix} \right]=\frac{B}{2}e^{-i2\theta}\left[ \begin{matrix} 1 \\ 0 \end{matrix} \right]$ (S7)

Here, the non-converted part is filtered out and the output vector is solely relative to the geometric phase, $-2\theta,$ for the incident RCP light beam.

**2.2 Incident linearly polarized (LP) light beam**

For the incident LP light beam, the QWP1 and QWP2 are removed. The Jones matrix of the LP light beam is given by $\left[ \begin{matrix} \cos\alpha\\ \sin\alpha\end{matrix} \right]$, where $\alpha$ is the polarized direction. When the LP beam generated by a linear polarizer P1 passes through a nanorod, the Jones vector can be written as

$J_{nanorod} LP=\frac{2A+B}{2}\cdot\left[ \begin{matrix} \cos\alpha\\ \sin\alpha\end{matrix} \right]+\frac{B}{2}\left[ \begin{matrix} \cos\left( 2\theta-\alpha\right) \\ \sin\left( 2\theta-\alpha\right) \end{matrix} \right]$ (S8)

There are two components coming out from the nanorod, a non-converted part: $E_{nclp}=\frac{2A+B}{2}\cdot\left[ \begin{matrix} \cos\alpha\\ \sin\alpha\end{matrix} \right]$ and a converted part: $E_{clp}=\frac{B}{2}\left[ \begin{matrix} \cos\left( 2\theta-\alpha\right) \\ \sin\left( 2\theta-\alpha\right) \end{matrix} \right]$.

**2.3 Filtering the non-converted part from the incident LP light beam**

After the non-converted part ($E_{nclp}$) passing through the polarizer P2, the output vector can be written as

$E_{nclp}^{out}=J_{P2}E_{nclp}=\left[ \begin{matrix} {cos}^{2} \delta& \sin\delta\cos\delta\\ \sin\delta\cos\delta& {sin}^{2} \delta\end{matrix} \right]\frac{2A+B}{2}\cdot\left[ \begin{matrix} \cos\alpha\\ \sin\alpha\end{matrix} \right]$ (S9)

To filter out the $E_{nclp}$, the angle $\delta$ have to be perpendicular to the angle $\alpha$. Thus, the $\delta$ can be $\alpha\pm\frac{\pi}{2}$. Therefore, the non-converted part can be filtered out as follows

$E_{nc}^{out}=\frac{2A+B}{4}\left\{ \left[ \begin{matrix} \cos\alpha\\ \sin\alpha\end{matrix} \right]+\left[ \begin{matrix} \cos\left( \alpha\pm\pi\right) \\ \sin\left( \alpha\pm\pi\right) \end{matrix} \right] \right\}=\left[ \begin{matrix} 0 \\ 0 \end{matrix} \right]$ (S10)

At this moment, the output vector of the converted part passing through the analyzer can be written as

$E_{clp}^{out}=J_{P2}E_{clp}=\frac{B}{4}\left\{ \left[ \begin{matrix} \cos\left( 2\theta-\alpha\right) \\ \sin\left( 2\theta-\alpha\right) \end{matrix} \right]+\left[ \begin{matrix} -cos \left( 3\alpha-2\theta\right) \\ -sin \left( 3\alpha-2\theta\right) \end{matrix} \right] \right\}$ (S11)

According to the predesigned polarization angle $\phi$, which is relative to the phase shift of $2\theta$ by the geometric phase. Thus, the Equation S11 can be modified to

$E_{clp}^{out}=\frac{B}{4}\left\{ \left[ \begin{matrix} \cos\left( \phi-\alpha\right) \\ \sin\left( \phi-\alpha\right) \end{matrix} \right]+\left[ \begin{matrix} -cos \left( 3\alpha-\phi\right) \\ -sin \left( 3\alpha-\phi\right) \end{matrix} \right] \right\}$ (S12)

From Equation S12, the darkest intensity captured by a CCD camera can be found at the position that $\phi-\alpha=3\alpha-\phi$. Thus, the darkest intensity or a gap of the 3D knots can be found at the generated polarization angle $\phi=2\alpha$.

**Supplementary Section 3. Metadevice to create five 3D-polarization knots with a single operation wavelength**

In this design, the 3D polarization structures are the knot 1 (a 3-foil knot; $\phi$ varies from 0 to 2π), knot 2 (a 4-foil knot; $\phi$ varies from 0 to 2π), knot 3 (a 3-foil knot; $\phi$ varies from π/4 to 9π/4), knot 4 (a 4-foil knot; $\phi$ varies from π/4 to 9π/4), and knot 5 (a 5-foil knot; $\phi$ varies from 0 to 2π). The parameter *a* is defined as 10 μm, $f_{0}$ is 500 μm, and *r* is 70 μm. The orientation angles $\Lambda_{m}$ of the knots are respectively 0°, 90°, 180°, 270° for knot 1, 2, 3, and 4, while the knot 5 is designed to be on-axis propagation (see Figure S2a). Their polarization profiles are represented as yellow arrows. The metadevice is designed to generate five 3D polarization knots at a single operation wavelength of 650 nm on the same observation region. The calculated phase profile and the SEM image of the fabricated metadevice are shown in Figure S2b and S2c, respectively.


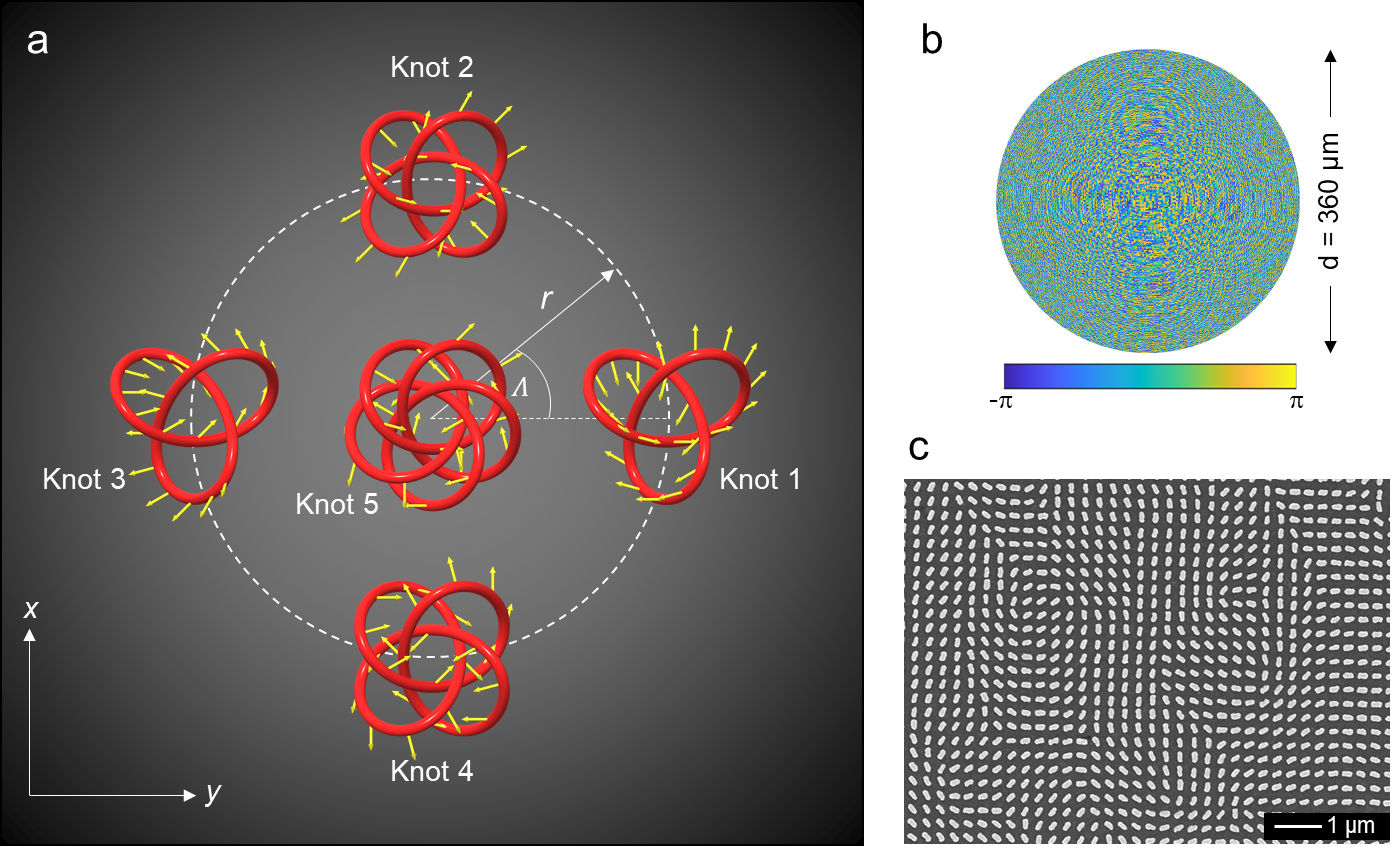


**Figure S2.** (a) An orientation of the 3D knots with a predesigned polarization profile (yellow arrows) in the *xy* plane. (b) The phase profile and (c) the SEM image of the fabricated metadevice for creating the five 3D polarization knots for an operating wavelength of 650 nm.

Figure S3 shows simulation and experimental results of the created five 3D polarization knots for a single operating wavelength of 650 nm at three observation planes (*z* = 485 μm, 500 μm and 515 μm). With the incident LP light beam, the gaps can be found on those three observation planes when the transmission axes of the P1 and P2 are (45°, 135°), (0°, 90°), and (75°, 165°), respectively (Figure S3b). The experimental results are in good agreement with the simulated results.

**
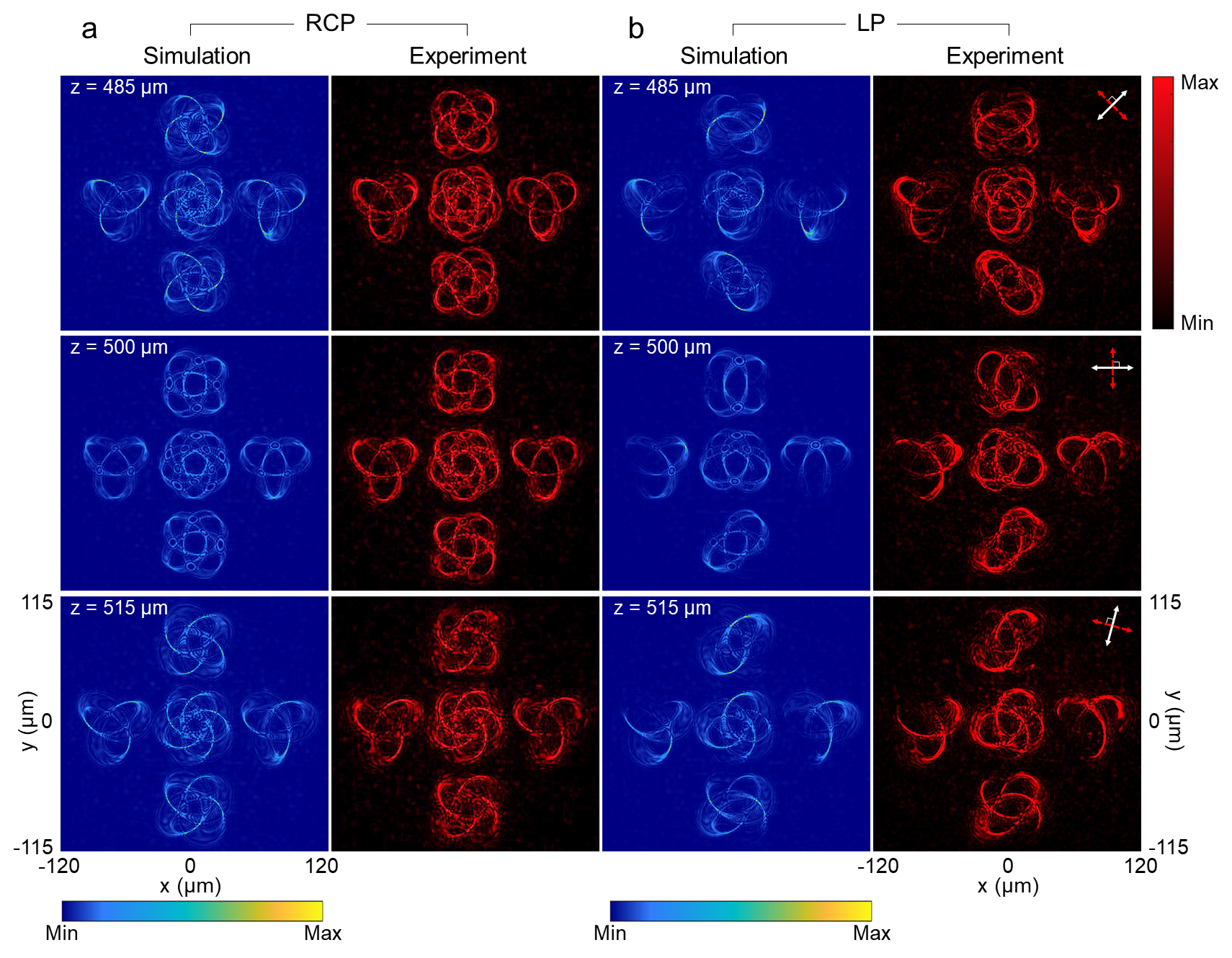
**

**Figure S3.** Simulation and experimental results of the created five 3D polarization knots for a single operating wavelength of 650 nm. (a) The intensity distribution at different observation planes under the illumination of RCP light. (b) The intensity distributions under the illumination of a LP light beam for polarization distribution detection with different transmission axes of the linear polarizer and the analyzer at different detection planes. The white and red double arrows indicate the transmission axes of the first polarizer (P1) and the analyzer (P2); P1⊥P2. The direction of the white arrows: 45° (*z* = 485 μm), 0° (*z* = 500 μm), and 75° (*z* = 515 μm) w.r.t. the *x* axis.

**Supplementary Section 4. Locations of created 3D knots at different wavelengths**

Figure S4-S6 show the locations of three different 3D knots that can be found at the different observation regions for the color selective metadevice. When the metadevice is illuminated by RCP light at *λ* = 650 nm (red), three polarization knots are generated (Figure S4). The 5-foil knot can be found at the region between *z* = 365 and 389 μm. The 4-foil knot can be found at the region between *z* = 425 and 452 μm. The 3-foil knot can be found at the region between *z* = 485 and 515 μm. When the metadevice is illuminated by RCP light at *λ* = 575 nm (green) (Figure S5). The 5-foil knot can be found at the region between *z* = 418 and 444 μm. The 4-foil knot can be found at the region between *z* = 485 and 515 μm. The 3-foil knot can be found at the region between *z* = 552 and 586 μm. When the metadevice is illuminated by RCP light at *λ* = 500 nm (blue) (Figure S6). The 5-foil knot can be found at the region between *z* = 485 and 515 μm. The 4-foil knot can be found at the region between *z* = 562 and 596 μm. The 3-foil knot can be found at the region between *z* = 638 and 677 μm.


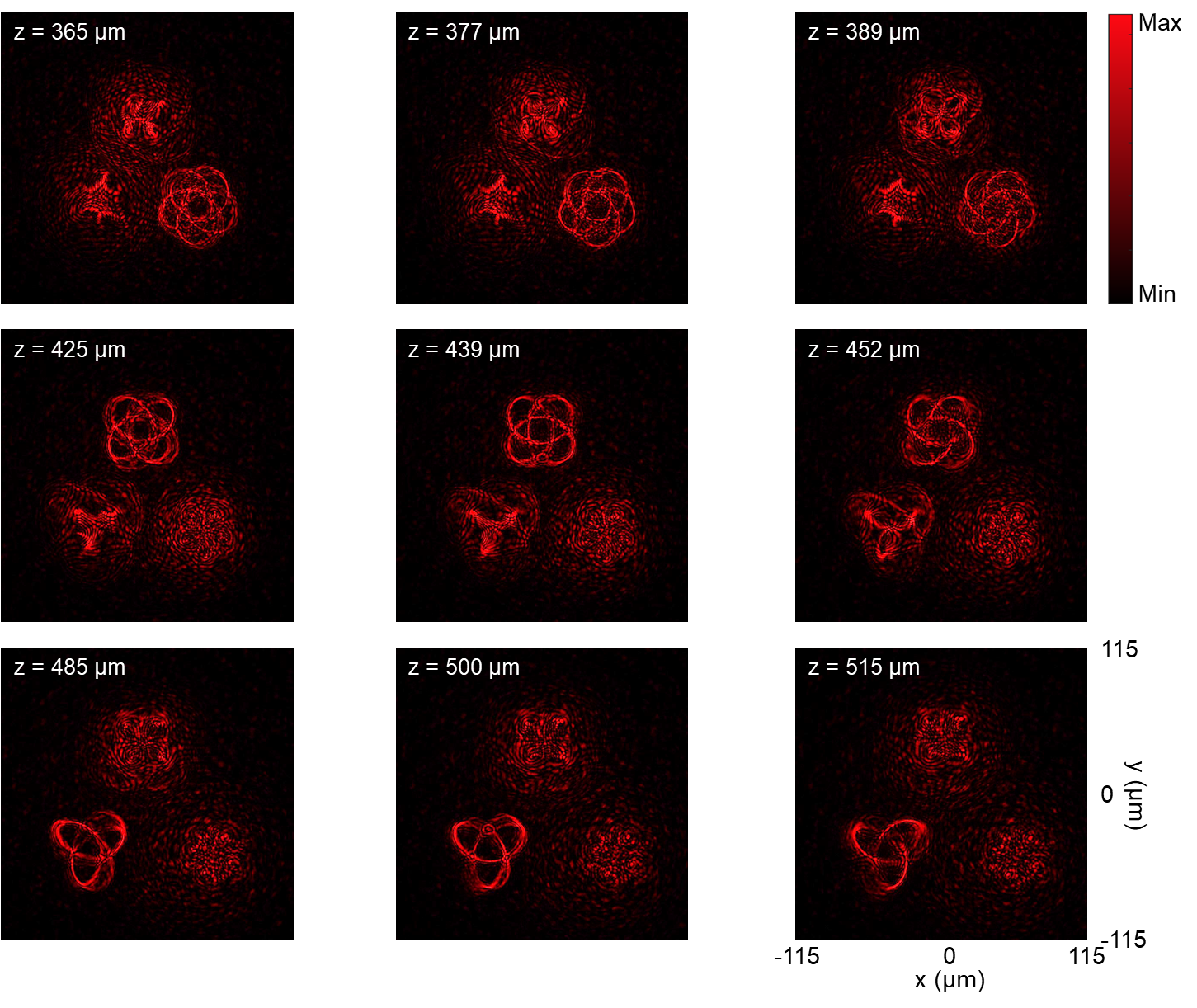


**Figure S4.** Locations of the created 3D knots upon the illumination of *λ* = 650 nm.


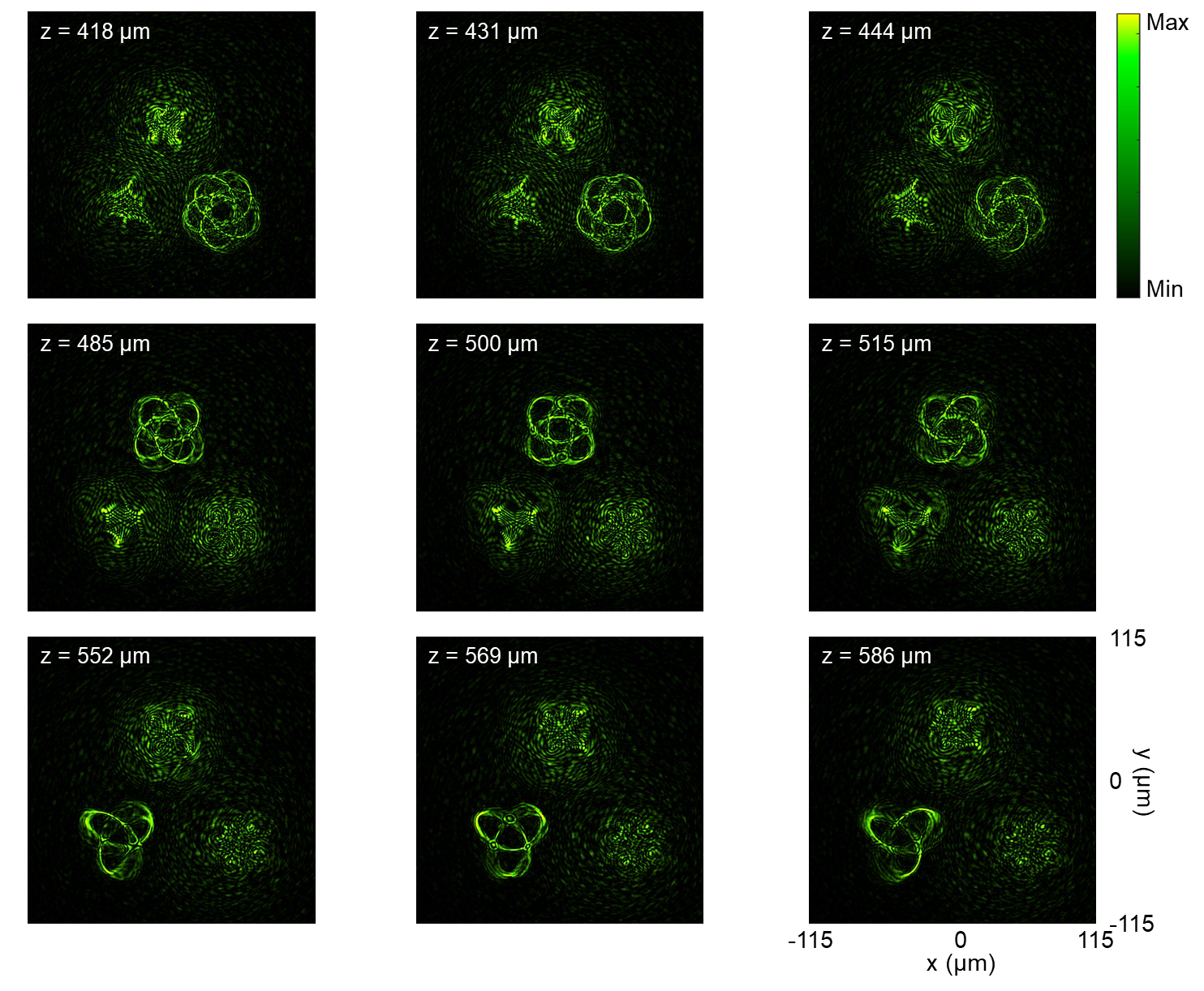


**Figure S5.** Locations of the created 3D knots upon the illumination of *λ* = 575 nm.


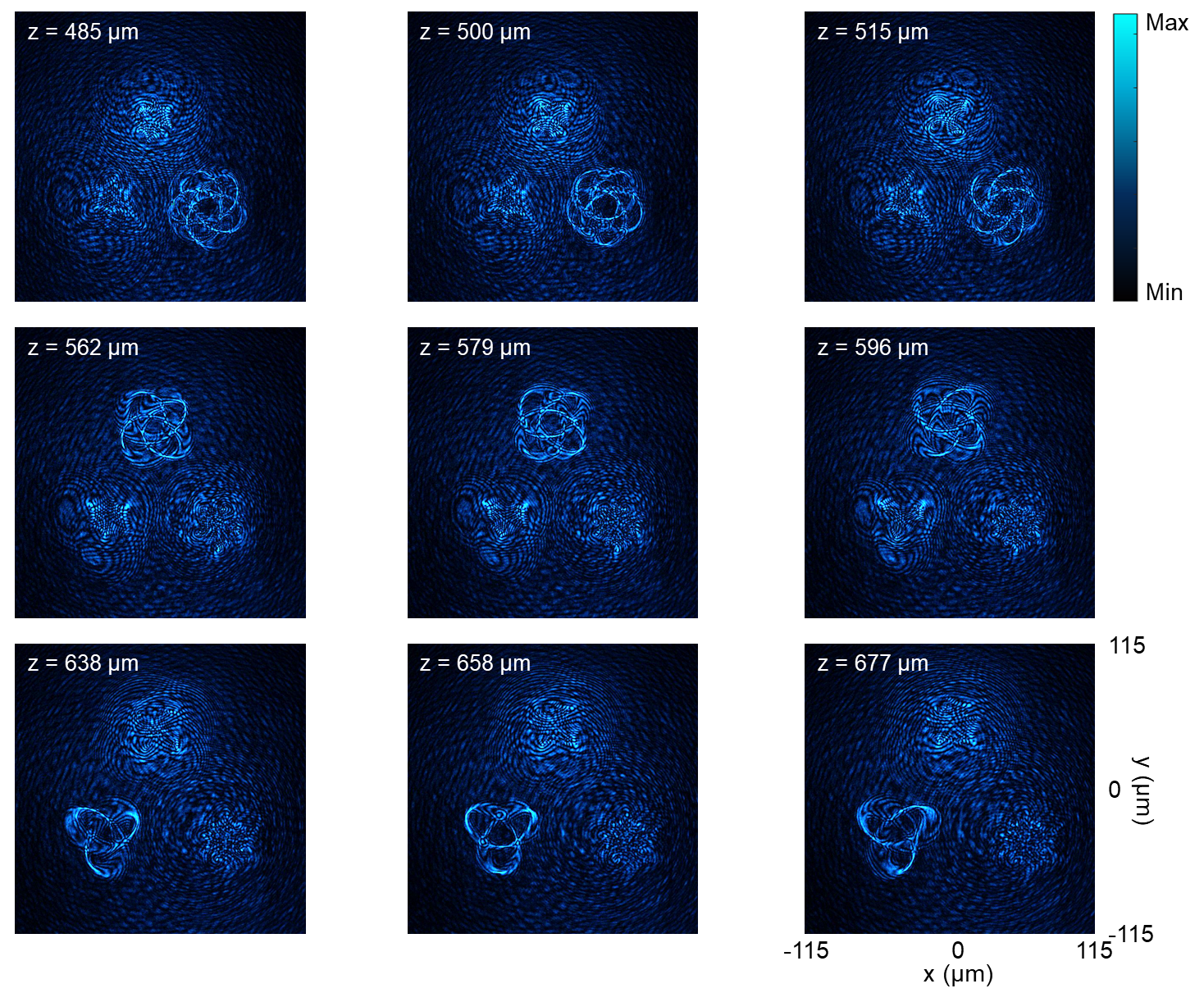


**Figure S6.** Locations of the created 3D knots upon the illumination of *λ* = 500 nm.

**Supplementary Section 5. Simulation results for other types of 3D knots**

The proposed method can be further extended to control other degrees of freedom by using another parametric equation for the generation of 3D knots, which are given by,

$\left\{ \begin{aligned} u_{m,n}=a\cos\left[ p\phi_{m,n} \right](1.8+\cos\left[ q\phi_{m,n} \right])+r\cos\Lambda_{m} \\ v_{m,n}=a\sin\left[ p\phi_{m,n} \right]\left( 1.8+\cos\left[ q\phi_{m,n} \right] \right)+r\sin\Lambda_{m} \\ f_{m,n}=-a\sin\left[ q\phi_{m,n} \right]+f_{0} \end{aligned} \right.$ (S13)

Where $r\cos\Lambda_{m}$ and $r\sin\Lambda_{m}$ are the locations of the knot *m* in the *xy* plane with a radius of *r* from the center of a focal plane and an angle of $\Lambda_{m}$ with respect to (w.r.t.) the *x* axis (Figure S7a). The types of knots can be defined by the integer numbers *p* and *q*, which are the times around its axis of rotational symmetry and that around a circle in the interior of the knot, respectively^3,4^. By controlling *p* and *q*, different (*p*, *q*)-knots can be generated. *a* is a constant number used to define the knot dimensions. The polarization direction $\phi_{m,n}$ of any point *n* on the knot *m* is denoted with a yellow arrow. The plane ${z=f}_{0}$ is the middle observation plane of the 3D knots.

In the design, *a,* $f_{0}\mathrm{and}r$ are 15 μm, 500 μm and 50 μm, respectively. The orientation angles $\Lambda_{m}$ of the knots are 210°, 90°, and -30° for *m* = 1, 2, and 3, respectively. The polarization rotation angles of the points on each knot vary from 0 to 2π. In order to maintain the same distance between two adjacent points for different knots, the knots are designed to have different numbers of the total points *N*, which are 2000, 2860 and 3728 points for the knot 1, 2, and 3, respectively. The knot 1, 2, and 3 are the (2, 3)-knot, (3, 4)-knot, and (2, 5)-knot, respectively. The simulation results are shown in Figure S7b for the incident RCP light at the wavelengths of $\lambda_{1}$ = 650 nm, $\lambda_{2}$ = 575 nm, and $\lambda_{3}$ = 500 nm, respectively.


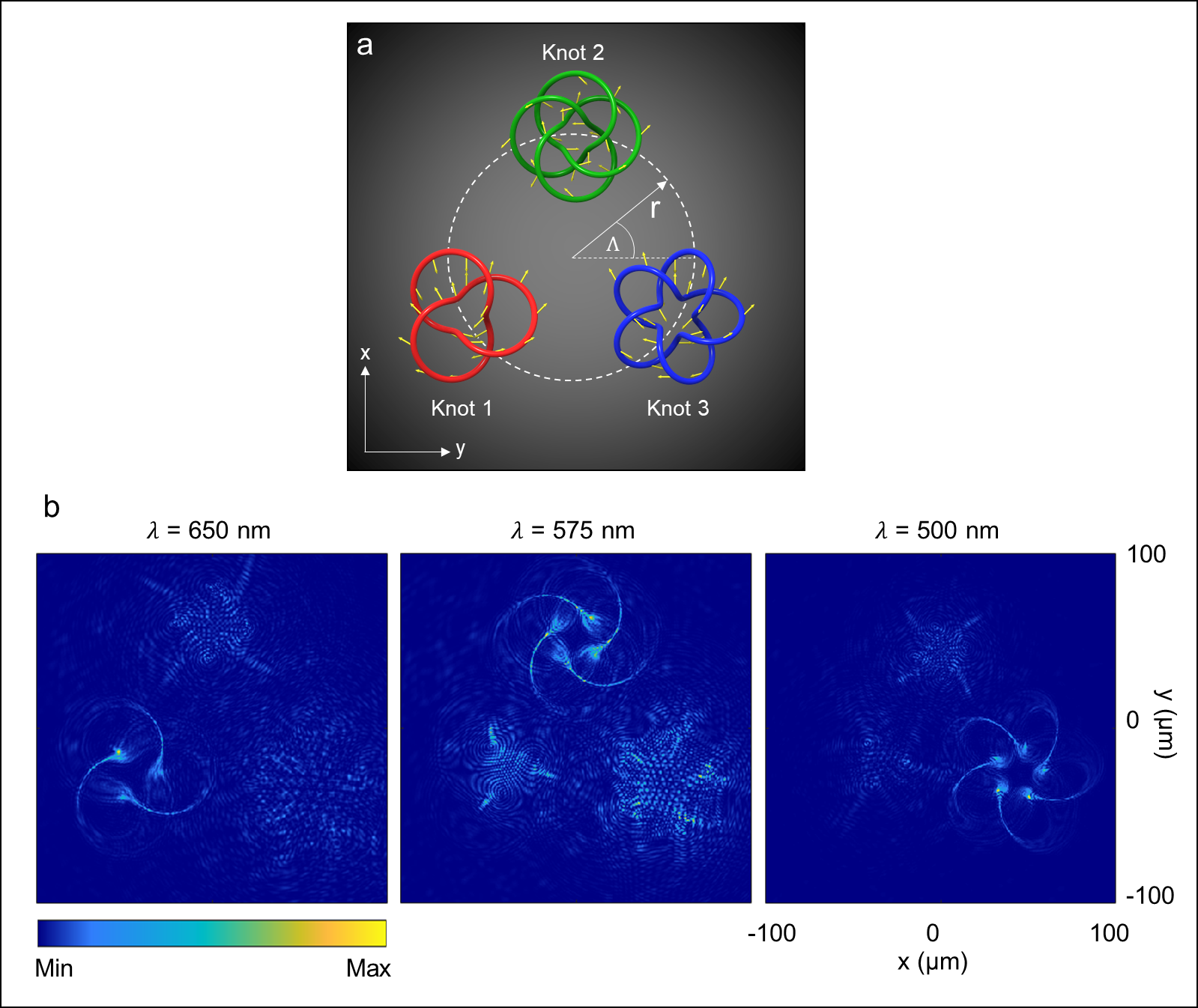


**Figure S7** (a) The arrangement of the 3D polarization knots. The polarization direction of any given point is denoted with yellow arrows. (b) The simulation results of the generated 3D polarization knots at $\lambda_{1}$ = 650 nm, $\lambda_{2}$ = 575 nm, and $\lambda_{3}$ = 500 nm on the observation plane of z = 515 μm.

**Supplementary Section 6. The same polarization knot with different color and polarization distributions**

In this design, Equation 6 in the main text is used to design the 3-foil knot with the combination of three different colors (*M* = 3; *r* = 0; $a=$ 10 μm; $f_{0}=$ 800 μm) as show in Figure S8a and S8b. In this case, the predesigned polarization profiles of three colors are different from each other. Each color has 700 focal points (*N* = 700), meaning there are 2100 focal points in total. The focal points 1 to 700 are encoded with the wavelength of 650 nm (*m* = 1), where the polarization rotation distribution is $15\phi_{m,n}$ (linear relation). The focal points 701 to 1400 are designed for the wavelength of 575 nm (*m* = 2), where the predesigned polarization profile is $2\phi_{m,n}^{2}$(nonlinear relation). The focal points 1401 to 2100 are designed for the wavelength of 500 nm (*m* = 3), where the predesigned polarization profile is $\phi_{m,n}-2\pi/3$ (different linear relation). In the design, the $\phi_{m,n}$ is changed linearly from 0 to $2\pi$ with the change of the focal points from 1 to 2100 (Figure S8c). The simulation results for the incident RCP light beams at the wavelengths of 650 nm, 575 nm, and 500 nm are shown in Figure S8d. The simulation results for the incident LP light beams along the *x* axis and the transmission axis of the analyzer along the *y* axis are shown in Figure S8e. Here, the gaps can be found at the positions where the predesigned polarization rotation is equal to $2$ and $2+$ (*α* is the polarization direction of the incident LP light beam w.r.t. the *x* axis).


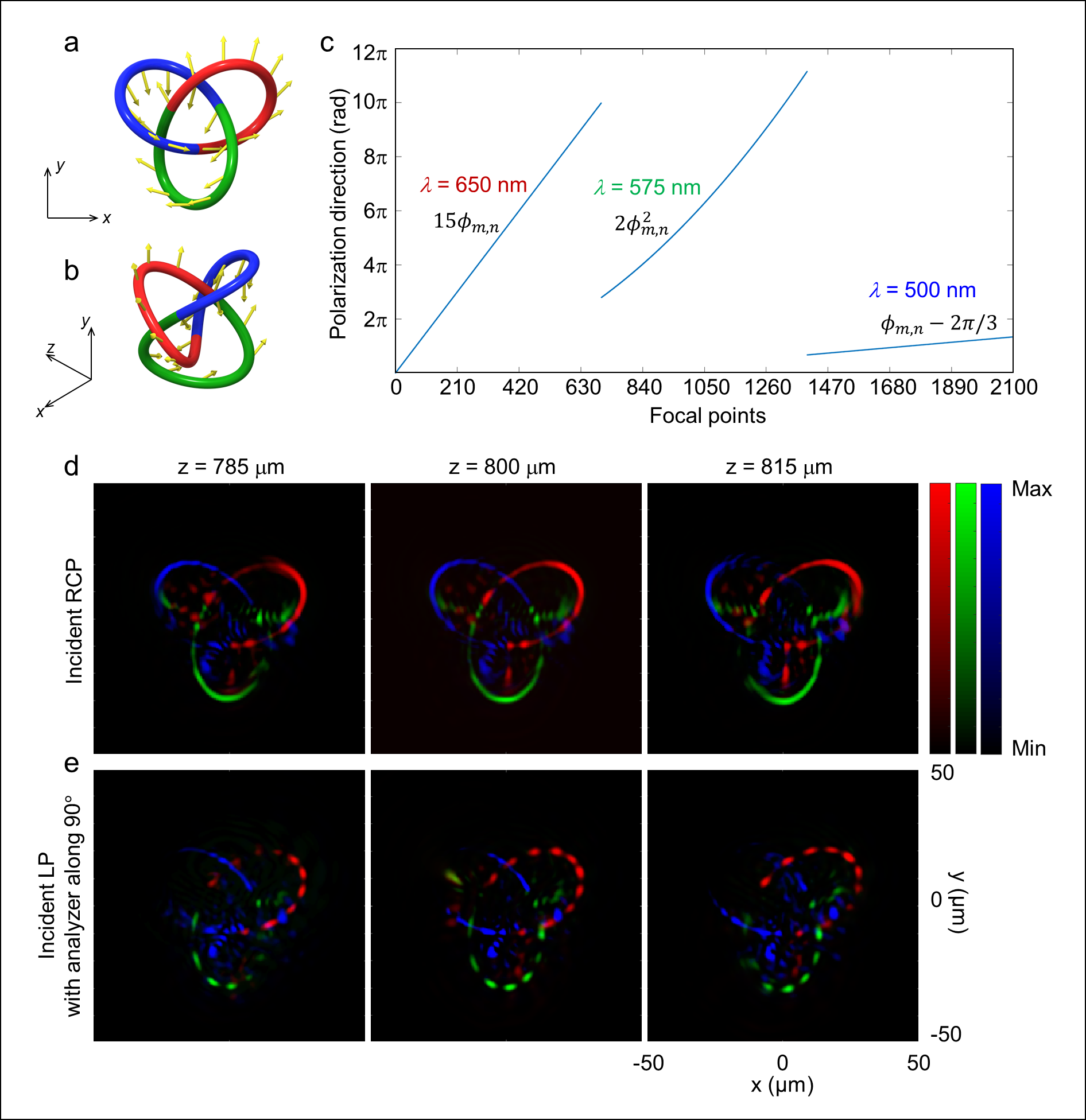


**Figure S8** (a) The front and (b) side views of the 3D knot with different color and polarization distributions on the same knot. (c) The relation between the polarization rotation angles and the focal points. (d) The simulation results of the intensity distributions at different observation planes under the illumination of RCP light beams and (e) LP light beams for the wavelengths of 650 nm (red), 575 nm (green), and 500 nm (blue).

**References**

1 Wang, R. *et al.* Metalens for Generating a Customized Vectorial Focal Curve. *Nano Lett.* **21**, 2081-2087 (2021).

2 Intaravanne, Y. *et al.* Phase Manipulation-Based Polarization Profile Realization and Hybrid Holograms Using Geometric Metasurface. *Adv. Photonics Res.* **2**, 2000046 (2021).

3 Murasugi, K. in *Knot Theory and Its Applications*, (ed Kunio Murasugi) Ch.7 (Birkhäuser Boston, 1996).

4 Thormählen, T. GSN Composer https://www.gsn-lib.org/docs/nodes/MeshTorusNode.php (2022).
